# Resurfacing Versus Non-resurfacing Patella in Total Knee Replacement: When and What to Choose

**DOI:** 10.7759/cureus.44276

**Published:** 2023-08-28

**Authors:** Mohamed Samih, Hajar Fadili, Aniss Chagou, Abdeloaihab Jaafar, Bouchaib Zryouil

**Affiliations:** 1 Orthopedics and Traumatology, Cheikh Khalifa Bin Zayed Al Nahyan Hospital, Mohammed VI University of Health Sciences (UM6SS), Casablanca, MAR; 2 Neurology, Cheikh Khalifa Bin Zayed Al Nahyan Hospital, Mohammed VI University of Health Sciences (UM6SS), Casablanca, MAR; 3 Orthopedics and Traumatology, Faculty of Medicine, Mohammed VI University of Health Sciences (UM6SS), Casablanca, MAR

**Keywords:** knee osteo-arthritis, patellar denervation, patella resurfacing, patellar resurfacing, pain after total knee arthroplasty, anterior knee pain, patella surgery, total knee replacement complications, tka (total knee arthoplasty), total knee replacement (tkr)

## Abstract

Introduction: The opinion on the necessity of patella resurfacing has been deeply divided in the scientific community. There are three approaches to the management of the patella in total knee arthroplasty (TKA). The first method involves always resurfacing the patella, the second method involves never resurfacing it, and the third method involves resurfacing the patella only under specific circumstances. Different data support each of these strategies, but no consensus on the best management has been reached.

Methods: This is a retrospective study of 106 cases of TKA (including 29 resurfaced patellas and 77 non-resurfaced patellas), over 5 years, at the Trauma-Orthopedics Department of the Cheikh Khalifa International University Hospital, Mohammed VI University of Health Sciences, Casablanca, Morocco. Our objective is to conduct a comparative study between TKA with patella resurfacing and those without resurfacing, by analyzing the functional and radiological results obtained after the operation, to show each technique's advantages and disadvantages.

Results: The average age of our patients was 65 years with extremes ranging from 46 to 80 years. There was a clear female predominance with a sex ratio of 0.2. The left side was predominantly affected. Primary gonarthrosis was the main diagnosis. Our results showed no significant difference between the two groups in terms of patient satisfaction and Knee Society Score (KSS). Nevertheless, complications generated by resurfacing, such as infection, anterior pain, stiffness, and loosening of the patellar component, as well as additional costs and increased operative time, were observed. In the majority of meta-analyses, there was no discernible difference in clinical and functional results between the resurfaced and non-resurfaced groups, suggesting that patellar resurfacing is not beneficial and, therefore, unnecessary.

Conclusion: It appears that regular resurfacing is not necessary. However, there is agreement that resurfacing the patella is the best course of action for patients with inflammatory arthropathy, considerable patella malalignment, and severe patellofemoral osteoarthritis. There are arguments in favor of each of the patella resurfacing techniques, but none of them are particularly compelling, each of these strategies has its advantages and should not be considered bad. The final decision, therefore, rests on each surgeon's practice, training, and experience.

## Introduction

Total knee arthroplasty (TKA)​​​​​​ corresponds to the prosthetic replacement of the femorotibial and patellofemoral compartments by an inert mechanical structure, allowing it to regain mobility and indolence, to recover its previous autonomy [1]. TKA has evolved, and this evolution has improved the implants and the friction torques to ensure better survival and longevity of the prosthesis. Early TKA designs were associated with a high level of anterior knee pain, as they did not take into account the patellofemoral joint. Patellar resurfacing in TKA was gradually introduced in the 1970s and was heralded as the savior of patient satisfaction due to the reduction in anterior pain [2]. Subsequently, systematic replacement of the patellar surface became part of the surgical strategy during TKA [3]. Recent designs, including patella replacement, have been able to reduce the incidence of anterior knee pain. This method does, however, come with certain new risks, such as infection, patella fracture, extensor mechanism disruption, loosening of the patellar component, and patellar instability [4].

The opinion on the necessity of patella resurfacing has been deeply divided in the scientific community. According to the literature review, there are three approaches to patella management. The first method involves always resurfacing the patella, the second method involves never resurfacing it, and the third method involves resurfacing the patella only under specific circumstances. Different data support each of these strategies, but no consensus on the best management has been reached [5].

This work consists of a retrospective study of 106 TKA cases (including 29 resurfaced patellas and 77 non-resurfaced patellas), over 5 years, at the Trauma-Orthopedics Department of the Cheikh Khalifa International University Hospital, Mohammed VI University of Health Sciences, Casablanca, Morocco. The aim of this study is to carry out a comparative study between TKA with patella resurfacing and those without patella resurfacing, analyzing functional and radiological results obtained after the operation, showing the advantages and disadvantages of each technique, and to evaluate the functional and radiological results of our study series and compare them with the literature.

## Materials and methods

This is a retrospective descriptive comparative study, carried out on 106 knees from 100 patients, who received TKA (including 29 resurfaced patellas and 77 non-resurfaced patellas), in the Trauma-Orthopedics Department of the Cheikh Khalifa International University Hospital, Mohammed VI University of Health Sciences, Casablanca, Morocco. The period considered for this work is 5 years, from January 2017 to January 2022. The minimum period of follow-up is 1 year, and the mean follow-up was 18 months. 

Patients who underwent TKA (with or without patella resurfacing) were included: 96 cases presented a primary osteoarthritis, 10 cases had a secondary osteoarthritis, of which 5 cases were of inflammatory origin and 5 cases of post-traumatic origin. However, we excluded from this study, revision TKA, a history of knee septic arthritis, malignant knee tumors, previous patellectomy, patients whose records are unexploitable and patients lost to follow-up or deceased knowing that no link can be established between their death and the arthroplasty performed.

We recorded the preoperative variables based on information and parameters taken from the patient's medical records and all recorded on pre-established evaluation sheets, including gender, age, body mass index (BMI), medical and surgical history, inflammatory joint diseases and trauma history, the affected side, whether the gonarthrosis was primary or secondary, the surgical technique, radiological assessment (knee radiograph, the anterior/posterior view and lateral view, and a pangonogram), as well as clinical assessment using the clinical Knee Society Score (KSS).

After summoning all patients for postoperative clinical and radiological evaluation, and looking for potential complications of the surgical technique, we finally evaluated the overall functional outcome. All data were statistically analyzed using Excel software and Jamovi software version 1.6.23 (The Jamovi Project, Sydney, Australia). The patients provided written informed consent regarding the publication of this case. The data's confidentiality and anonymity were protected.

## Results

In a population of 100 patients (106 knees), the mean follow-up was 18 months. The average age of our patients was 65 years with extremes of age between 46 and 80 years. There was a predominance of women, 83 women (83%) and 17 men (17%). Thus, there was a predominance of women with a sex ratio of 0.2. The mean BMI of the patients was 32 kg/m2 with extremes ranging from 17 kg/m2 to 44 kg/m2. We noted 6 bilateral TKAs (5.7%), 54 were placed on the left (50.9%), and 46 on the right (43.4%). Concerning the etiologies of gonarthrosis, 96 cases (90.6%) presented primary senile osteoarthritis, and 10 cases (9.4%) had secondary osteoarthritis, of which 5 cases (4.7%) were of inflammatory origin and 5 cases (4.7%) of post-traumatic origin.

All patients received the same type of prosthesis, semi-constrained posterior stabilized. The classic approach, which is the medial parapatellar approach, was performed in 95 cases (89.6%). The lateral approach, known as the Keblish approach, was used in 11 cases (10.4%) of genu valgum deformity. Regarding patella management, 73% of patellas were not resurfaced and 27% of patellas were resurfaced. Denervation of the patella was performed in all of our patients; however, regularization and removal of osteophytes were performed in 42% of cases.

Referring to postoperative complications (Table 1), we noted in the resurfaced group 5 cases (17.2%) of anterior pain, 4 cases (13.7%) of infection, 2 cases (6.8%) of limping, and 1 case (3.4%) of stiffness, loosening, unchanged walking distance, and limited flexion. For the non-resurfaced group, we noted\ 3 cases (3%) of infection, 12 cases (15.5%) of anterior pain, 5 cases (6.4%) of limping, 2 cases (2.5%) of malalignment, and 1 case (1.2%) of unchanged walking distance, limited flexion, and clunk syndrome. No fractures, dislocations, patellar instability, or rupture of the extensor mechanism were noted in either group.

**Table 1:** Postoperative complications. n: Number of patients.

Postoperative complications	Resurfacing group (n=29)		Retention group (n=77)
Infection	5 (17.2%)		12 (15.5%)
Anterior pain	4 (13.7%)		3 (3%)
Limping	2 (6.8%)		5 (6.4%)
Loosening of the patellar component	1 (3.4%)		0 (0%)
Stiffness	1 (3.4%)		0 (0%)
Limited walking distance	1 (3.4%)		1 (1.2%)
Limited flexion	1 (3.4%)		1 (1.2%)
Malalignment	0 (0%)		2 (2.5%)
Clunk syndrome	0 (0%)	1 (1.2%)	
Patella fracture	0 (0%)	0 (0%)	
Extensor mechanism ruptures	0 (0%)	0 (0%)	
Patellar instability	0 (0%)	0 (0%)	

As for functional results (Table 2), regarding joint mobility, the average postoperative flexion of our patients increased from 82° preoperatively to 108.5° postoperatively, with an average of 108° in resurfaced cases, and 109° in non-resurfaced cases. Concerning the global KSS, the average score postoperatively was 163 instead of 83 preoperatively; specifically, 161.8 in the non-resurfaced group and 164.2 in the resurfaced group. And finally, anterior knee pain decreased from 76% of cases preoperatively to 11% of cases postoperatively. Concerning pain, we noted the total disappearance of pain in 85 cases (80%). Anterior knee pain decreased from 81 cases (76.4%) preoperatively to 7 cases (6.6%) postoperatively, including 4 cases of resurfaced patella and 3 cases of non-resurfaced patella.

**Table 2:** Postoperative functional evaluation. n: Number of patients. KSS: Knee Society Score.

Group	Resurfacing group [n= 29]	Retention group [n= 77]	All patients [n= 106]​​​​​​​
Postoperative KSS	164.2	161.8	163
Postoperative flexion	108°	109°	108.5°

## Discussion

In our study, the mean follow-up was 18 months, which is close to that of other series [6], and the indication for surgery was gonarthrosis, which is identical in all series. Concerning the mean age, we notice a concordance between our series and most series [7-8]. The high prevalence of gonarthrosis in this elderly population is related to the fact that advanced age is an important etiological factor in gonarthrosis [2]. Among the risk factors for gonarthrosis, age is the most important. Loeser et al. explain the relationship between aging and the development of osteoarthritis, oxidative stress disrupts specific cell signaling pathways, affecting the ability to maintain the extracellular matrix of cartilage and eventually leading to cell death, resulting in cartilage degeneration and subsequent primary gonarthrosis [9]. It has been shown that women are more severely affected by knee osteoarthritis. We note a similarity between our series and those in the literature. The cause may be multifactorial and include anatomical differences, and genetic and hormonal problems [10].

Traumatic knee injuries are common in young adults and contribute strongly to the premature and rapidly progressive development of knee osteoarthritis. Young patients expect to remain physically active; therefore, post-traumatic knee osteoarthritis poses a therapeutic dilemma for the physician, as no treatment is reasonably accepted by young patients who will ultimately be faced with the decision to undergo surgery, despite problems with the durability of the prosthesis, or to continue with ineffective non-surgical treatment [11]. This history is important to consider, as it influences the indications for TKA, the choice of technique, and surgical planning [1].

One of the most frequent reasons for ongoing issues following TKA is anterior knee pain. Patients may experience it with or without patellar replacement. The surgical process itself can result in some alterations that may impact how the patellofemoral joint's partners connect [12]. The incidence of anterior knee pain was reduced in the group that underwent resurfacing in four meta-analyses [13]. Furthermore, anterior knee pain is still a common complaint in patients undergoing patellar resurfacing and it has been suggested that patellar resurfacing should not be performed routinely [14], as this pain does not always disappear with secondary patella resurfacing [15]. Fuchs et al. found that patella resurfacing altered patellofemoral kinematics compared to the native knee, and thus may be related to anterior knee pain [16]. There is no strong evidence that the non-resurfaced patella generates anterior knee pain and it is well-known that there are multiple causes for anterior knee pain after TKA [5].​​​​​​

According to Fleaca et al., there are several complications associated with patella resurfacing, including patella fracture, infection, patellar tendon injury, avascular necrosis of the patella, instability requiring reoperation, and loosening of the patellar component [5]. There is a close relationship between the patellar resurfacing technique and the occurrence of these potential complications [17]. It has been shown that a residual patella bone thickness of less than 12 mm increases the risk of a stress fracture. Conversely, it appears that leaving too much patella thickness results in a higher risk of pain, subluxation, or loosening due to increased stress on the isolated patellofemoral joint [18]. 

In the non-resurfacing group, clunk syndrome, malalignment, and hematoma were identified as complications, the latter two of which are not related to the patellofemoral joint, nor to whether or not the patella resurfaced. In a systematic review, Sequeira et al. showed that the relationship between patellar resurfacing and the incidence of patellar clunk syndrome has not been definitively confirmed in the literature [19].

Studies found that there is no significant difference between patella resurfacing and non-surfacing in TKA concerning functional outcomes, KSS, Oxford Knee Score, Visual Analogue Scale, range of motion, and patient satisfaction. Therefore they questioned the effectiveness of routine patella resurfacing, suggesting that patella resurfacing is not beneficial, and therefore not necessary [14-20].

The limitation of this study is that the TKAs were not performed by the same surgeon, as the postoperative results also depend on the experience of each surgeon, which may create a confounding bias in the study.

## Conclusions

Patella replacement leads to higher expenses and a lengthened recovery period. In terms of clinical and functional outcomes, the majority of meta-analyses have not discovered a significant difference between the resurfaced and non-resurfaced groups. Therefore, systematic resurfacing is not always required. Inflammatory arthropathy, considerable patellar malalignment, and severe patellofemoral osteoarthritis are the main indications for resurfacing the patella, according to the consensus. There are reasons in favor of each method. Each of these methods has its benefits and should not be banished. The choice is ultimately determined by each surgeon's expertise and skills.
